# Understanding non-return after a temporary deferral from giving blood: a qualitative study

**DOI:** 10.1186/1471-2458-12-1063

**Published:** 2012-12-10

**Authors:** Tessa L Hillgrove, Kathleen V Doherty, Vivienne M Moore

**Affiliations:** 1 Discipline of Public Health, University of Adelaide, Adelaide, South Australia; 2 Australian Red Cross Blood Service, Adelaide, South Australia; 3 University of Tasmania, Hobart, Tasmania

## Abstract

**Background:**

The reasons why deferral from blood donation reduces the likelihood of future return remain unclear. This aim of this study was to investigate possible reasons why deferral has such a dramatic impact on donation patterns.

**Methods:**

Qualitative methods were used to explore donors’ motivations to give blood, their experiences of temporary deferral, and their intentions to return once eligible. Semi-structured interviews were conducted with 23 donors in the two weeks following a temporary deferral due to a low haemoglobin concentration. The Framework approach was used to analyse data and identify themes associated with prompt return, ascertained from Blood Service records.

**Results:**

We found that, predominantly, individuals give blood because it represents an easy and convenient way to help others, and provides personal rewards, such as enhancing positive self-concepts and valuable knowledge about health. Deferral disrupts the habit of regular donation, and additionally, introduces an element of practical and emotional hassle to what is generally seen as an undemanding activity. Return after deferral was related to four aspects of a person and their context: an individual’s other obligations, especially parenting; whether donation arrangements were facilitated by a range of supports; the presence of a strong “blood donor” identity; and whether deferral left the donor feeling valued and appreciated.

**Conclusions:**

Aspects of the deferral process need to be improved to ensure individuals feel valued, and continued attention should be given to the convenience of donation, especially for those with competing obligations.

## Background

Because only 3% of the Australian public donate, the Australian blood supply is reliant on a small group of committed, regular volunteer donors, making both recruitment and retention efforts vital to guarantee that the blood supply is maintained [1,2]. Occasionally, donors may be deferred from giving blood for reasons relating to their health and lifestyle, with the most common deferral due to a low haemoglobin concentration, affecting around 5% of donors each year [3]. During the pre-donation interview, a finger-prick blood sample is taken to measure a donor’s haemoglobin concentration. Those who fail to meet the minimum acceptable haemoglobin concentration are not eligible to give blood on the day, and, subject to the results of serum ferritin testing, are deferred from giving blood for a six month period.

Several studies have shown that donors are less likely to return to donate blood after a temporary deferral [4-9]. It is possible that some donors misinterpret their temporary deferral as being permanent [10], and that medical ineligibility, real or imagined, may result in self-deferral [11]. Donors who originally attended under the influence of social pressure may consider themselves “off the hook”, those with altruistic motivations may feel rejected and disappointed [7], and others are possibly annoyed at having their time wasted [8]. Deferred donors are more likely than non-deferred donors to say that donation is difficult, and report bad feelings after their experience [11]. Breaking the habit of donation may also play a role, as habitual behaviours are easily maintained in stable circumstances but must return to more conscious control in the face of a novel situation [12].

While several studies have investigated the influence of temporary deferral on subsequent donation patterns, none have specifically explored the reasons why deferral has such a negative impact on return. In order to understand why donors are less likely to return after a deferral, we conducted a qualitative study exploring donors’ motivations for giving blood, perceptions of the deferral experience, previous experiences of lapsing from donation, their intentions to return in the future, and actual return behaviour once eligible. We then developed a conceptual framework to explain return after a temporary deferral.

## Methods

Ethics approval was obtained from the Human Research Ethics Committees of the University of Adelaide and the Australian Red Cross Blood Service (the Blood Service).

### Recruitment

In mid-2007, on a weekly basis for three months, donation records were extracted for all South Australian donors deferred for low haemoglobin during the previous week. Donors were selected based on a recorded haemoglobin concentration of <120 g/L for women, and <130 g/L for men (the acceptance thresholds at the time of the study). Potential participants were selected through purposive sampling, a non-probability sampling method that ensures that the sample shares key characteristics with the population in question [13]. Care was taken to invite both men and women, and people with varying lengths of time as a blood donor, to participate in the interviews in order to capture a broad range of donation experiences and views. Between 5 and 10 potential participants were selected each week from a confirmed deferral list. For feasibility, donors who resided more than one hour away from the central business district, or who did not speak English (identified in the donor database by the need for an interpreter) were not approached for an interview, as these conditions are estimated to apply to a very small proportion of individuals donating in the city.

Donors were initially sent an information letter and a follow-up phone call was made approximately three days after mailing the letter to discuss interest in participation. A total of 50 donors were sent letters of invitation and 29 agreed to schedule an interview. Data saturation, the point at which the no new themes emerged, was deemed to occur after 25 interviews [14].

### Data collection and analysis

Semi-structured interviews were undertaken to allow donors to express a diversity of views and to allow for the emergence of new issues. Interviews were conducted by TH, using a semi-structured interview guide. The topics covered are shown in the section (Topics covered in the interview guide). All participants signed a consent form and gave permission to have the interview recorded. The first four interviews were used to develop and pilot the interview guide, with a further 25 interviews completed during May and June of 2007. Interviews took place between 7 and 20 days from the date of the deferral and ranged from 22 to 54 minutes in length. Poor quality recording resulted in two interviews being unable to be transcribed and therefore unable to be analysed further (only minimal notes were taken during interviews). As a result, 23 interviews were available for analysis. Interviews were transcribed and pseudonyms used to protect the identity of the participants. The demographic and donation characteristics of these 23 participants are shown in Table 1.

**Table 1:** Demographic and donation characteristics of study participants

**Category**		**n=23**
SEX*	Male	6
	Female	17
AGE*	17-24	6
	25-39	1
	40-54	11
	55+	5
PREVIOUS DEFERRAL^	No	8
	Yes- for low Hb	12
	Yes- for other reason	3
NUMBER OF DONATION VISITS** (including deferral)	1 (deferred at first attempt)	1
	First return after long gap (the Blood Service database reported first attempt)	3
	2-3	1
	4-10	5
	11-20	3
	21-49	5
	50+	5
LIFE STAGE^	High school student	1
	University student	3
	Working	12
	Home duties (not in paid employment)	1
	Retired	6
CHILDREN ^	Yes- still living at home	7
	Yes- left home	3
	No	9
	Not stated	4

* Based on information in the Blood Service database.

^ Based on information revealed in the interview.

** Based on information in the Blood Service database, revised in accordance with information revealed in the interview (e.g. 3 donors were recorded in database as first time donors, when in fact they had returned after a break).

### Topics covered in the interview guide

What are donors’ perceptions of the deferral experience?

What do donors understand about the reasons for their deferral?

What are donors’ intentions regarding seeking further investigations into the cause of their low haemoglobin, and what is their motivation for doing so?

How do donors talk about their intentions to return once eligible?

Do participants see themselves as “blood donors”, and how do their self-perceptions compare with the concept of a “blood donor identity”?

Motivations for donating for the first time, and then for continuing to give blood

Descriptions of unsatisfactory donation experiences

The circumstances leading to a cessation from donation during previous phases of the donor career, and recommencement after the break

Reflections on giving blood as a voluntary activity

Responses to the information that deferral reduces the likelihood of return

Analysis was guided by the Framework approach [15], which was developed for use in applied policy research. The Framework approach is useful when research objectives are defined from the outset, such as explaining the findings of quantitative research, as it is geared towards supplying “answers” that address specific questions. The Framework approach has five distinct stages. The first three, *familiarization,* deriving a *thematic framework*, and *indexing* (coding the data against the thematic framework) are common to other qualitative methods. The final two stages are specific to the Framework approach. The fourth stage, *charting,* involves building a picture of the data as a whole, and looks to explain variation and identify patterns. The fifth and final stage, *mapping and interpretation* involves interpreting the data as a whole, in accordance with the key objectives set at the beginning of the study. Analysis was performed using NVivo 7 software [16].

After the indexing stage of analysis had been completed, whether or not donors had returned after being eligible to do so was ascertained from the Blood Service donor database. A cut-off of nine months from being eligible to return was selected as time-to-event analysis conducted previously revealed that donors were unlikely to return beyond this point if they had not already done so [4]. A flag was created in NVivo to indicate whether each donor returned or did not return within the specified time period, and comparisons were made between return status and themes identified in the coded interview data. Data were analysed primarily by TH, who met weekly with VM to discuss emerging themes and perform theoretical comparisons against existing literature.

## Results

The first part of the results section presents analysis of participants’ motivations for giving blood. The next part describes the circumstances leading to previous breaks from giving blood, and is followed by participants’ experiences of deferral and their intentions to return. Finally, we present analysis of the themes that were consistently linked with whether or not a donor returned once eligible, and a conceptual model resulting from this analysis.

### Motivations for giving blood, self-perceptions and benefits

When asked why they donated blood, participants described a range of motivations including altruism and an awareness of need, following the example set by parents and family members, and personal knowledge of a transfusion recipient. Several donors described the habitual nature of their commitment to give regularly.

“It’s not something that you really think about, it’s just something that you do once every three months” (Female, 18)

Most participants had thought about giving blood for a long period of time before their first attempt. Intentions were often translated into action after a specific trigger, such as encouragement from others, an organised group effort, a particular appeal from the Blood Service, or a convenient opportunity. For example, one participant described the reason she finally donated with a work group.

“Because it was organised for me…Because it was easy. Because I didn’t have to think” (Female, 24)

Some donors also described other ways that attending to give blood had been facilitated by supportive environments, including being allowed by their employers to give blood during work hours. However, the majority of participants did not appear to be supported this way.

The majority of participants saw themselves as a “blood donor” which was predominantly seen as eliciting positive self-perceptions, such as being unselfish, useful, and community-minded.

“I’m quite proud to say that I do it … I guess it just helps make you be the person you want to be” (Female, 26)

Giving blood was considered an appropriate activity for someone enjoying good health, and meeting the criteria to give blood reinforced an individual’s status as a healthy person.

“I’m fairly healthy, one regular partner…I’m in a good position. I should do it.” (Female, 45)

**Figure 1:**
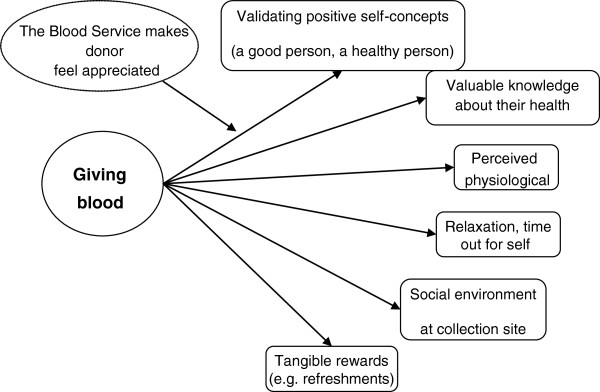
Personal benefits of being a blood donor.

Participants also described extrinsic and intrinsic benefits of giving blood (Figure 1), including positive self-concepts, feeling valued, and taking time out for themselves.

“They make you feel welcome. And they make you feel if you have done something really good and afterwards, they always sort of thank you for doing it…they sort of make you feel, how should I put it, special” (Female, 65).

Relatively few negative aspects of donation were reported. The most common drawbacks were the inconvenience of the time required to give blood, discomfort of the needle, and physical reactions to donation. These negatives tended to be described as minor inconveniences.

### Blood donation compared to other altruistic activities

Participants were asked to reflect on giving blood compared to other altruistic activities, such as volunteering time or donating money to a charity. Most described blood donation as a smaller investment of time and energy than volunteering, and a contribution that was possible regardless of financial situation, unlike donating money.

“You do feel as if you are helping out, cause even in all other aspects of life where you do selfish things …you can do something without having to give money or without having to give a lot of time and do things” (Female, 18)

Another appealing characteristic was that blood donation didn’t require a regular and sustained commitment, as donors gave as part of a pool. Correspondingly, postponing donation was perceived not to have catastrophic consequences.

“It’s not like you don’t go one day and everything’s going to crash down. Like you help when you can” (Male, 22)

### Reflections on ceasing donation and previous experience of “lapsing”

Participants were asked their views on ceasing donation. For the most part, deferred donors did not plan to stop giving blood, and most suggested that the decision would be imposed upon them. However, two of the longest serving donors noted that their continued commitment to giving blood depended on the activity remaining easy.

“I think if it was a big hassle you’d think twice, you know you’d think “oh I can’t be bothered”, and then, you stop doing it once or twice and then you’d probably get out of that habit and not go.” (Female, 64)

**Figure 2:**
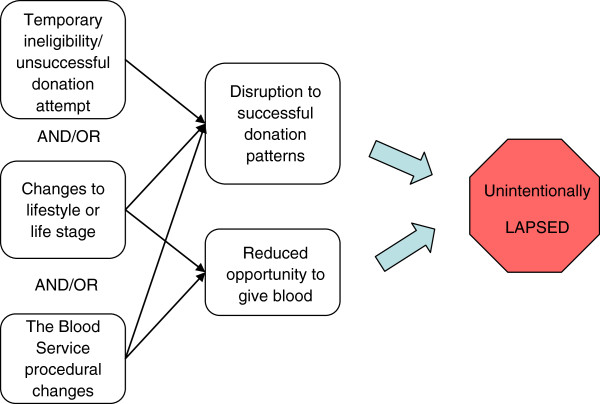
Pathway to unintentionally lapsing from donation.

Many participants discussed circumstances surrounding previous breaks from donation. There were a number of common themes in these accounts. First, there were two factors that contributed to a break: changes in personal circumstances (such as change in job, moving further from a collection site, or having children, see Figure 2); and/or changes in Blood Service policy or procedures that reduced the convenience of donation (such as a change in opening hours, mobile collection locations, or acceptance criteria). Second, breaks often began due to temporary ineligibility or an unsuccessful donation attempt, which happened to coincide with changes in personal circumstances. The combination of life events and decreased opportunity for donation resulted in breaks from which donors did not readily return. These donors could be thought of as “unintentionally lapsed” and this appeared to be particularly common amongst women who said they had dependent children. Crucially, donors did not describe any changes in their attitude towards giving blood leading up to the break, nor during the break itself. Rather, the habit of donation had been disrupted. One example is provided here.

“After the first time I got knocked back, I think it was 12 months that I couldn’t give blood, and you do kind of get out of the habit of it and then that’s the same time that it changed, and it moved, and so you had to make a conscious effort to always remember to go down , and sometimes you could sit there for an hour before you were actually seen, …so a couple of times it was inconvenient in that regard” (Female, 47).

Only one participant described intentionally delaying returning after an unsuccessful donation attempt unrelated to low haemoglobin. She initially stated the reason for slow return was *“laziness”*, however, when prompted she offered a different perspective, suggesting that she had deliberately avoided returning because she thought her donation might be refused.

Several donors returned from a substantial break after a specific trigger or prompt, similar to the reasons given by participants for instigating a first attempt. These triggers all occurred at a stage when donation could be more easily accommodated in their lives, although it appeared the circumstances had been favourable for a period of time prior to returning.

### Perceptions of the deferral experience

Most participants described negative emotional responses resulting from their deferral. Some strong negative emotions resulted from denial of the opportunity to help and the disruption to the donors’ self-perceptions as capable, competent, and healthy individuals. Some interviewees were also anxious about a possible underlying condition.

“I'm really, really upset at myself… the whole day, yeah it's a feeling of rejection and “how can you not be disciplined enough to eat the right foods” … I ring my mum, my sister and I say “I'm real sad, I've been rejected”” (Female, 44)

“Shocked, disappointed…just finding my iron was low when I felt so well… I wasn’t disappointed that the iron was low or anything, I was just disappointed about not being able to give” (Male, 49)

Others were annoyed at having their time wasted, or upset with the way they were treated by collection staff.

“[The collection nurse] was a bit sort of snappy …she didn’t upset me, not easily upset but it was a bit abrupt more than anything” (Female, 51)

Most negative emotional responses to deferral appeared to be short lived, and it was common for donors to say they hadn’t thought about their deferral much since the event. At the same time, some also recognised that they had gained valuable knowledge about their health (elaborated below). A small group reported no negative responses to deferral. Most of this group had been deferred for low haemoglobin on more than one occasion, and some had anticipated the most recent deferral event.

### Deferral and implications for health

The Blood Service provides information about haemoglobin, iron, and the purpose of further testing after deferral for low haemoglobin in three ways: information brochures available immediately following the deferral (the first containing information about the role of haemoglobin, iron, and ways to increase iron intake and absorption, and the second explaining the need for deferred donors to seek further testing and the possibility of conversion to apheresis donation); a verbal explanation from the collection nurse; and in a letter mailed to deferred donors with their Ferritin test result.

The letter regarding Ferritin test results was a specific source of confusion as not one donor was familiar with the word “ferritin” and several donors queried its meaning during the research interview, indicating the letter did not contain an adequate explanation.

Donors expressed poor understanding of haemoglobin in biomedical terms, with limited understanding of the role of haemoglobin in the body, and its relationship to dietary iron intake or possible underlying disease.

“I wouldn’t have any idea, I don’t know… I figured that it would have to be of some importance, it’s something to do with iron or something like that?” (Female, 18)

Although most donors had limited understanding about the role of haemoglobin in the body, it did not diminish their belief that there were justifiable reasons for their deferral, indicating high levels of trust in the organisation.

“I suppose there must be technical reasons why they defer low haemoglobin. In other words … who am I to question why.” (Male, 54)

While most donors expressed negative responses to their deferral, some also recognised that being deferred had benefits.

“It’s a blessing as well because if I didn’t go on Saturday and my iron count was low, who knows where that would have led to.” (Male, 49).

In relation to the verbal explanation provided by the collection nurse, some participants felt the reasons suggested, such as poor diet, stress, and heavy menstrual cycles, did not necessarily apply to them.

Three donors described interactions with nursing staff that were less than satisfactory, characterised by inadequate explanations and poor staff treatment.

“If they had just taken two minutes to say, “well it is a bit a low and this is why we don't want to take it”, I would have walked about thinking “oh fair enough” that would have been that. But I sort of thought, I had wasted an hour to be told nothing.” (Female, 41)

### Which personal characteristics and circumstances are associated with return

Only 11 of the 23 participants returned within nine months of being eligible to do so. One additional donor was not eligible to return (on medical advice), and one participant was deceased at the time of follow up. Four aspects of a person and his/her context were found to be associated with whether or not a donor returned promptly once eligible: being female with dependent children, having donation facilitated by a supportive environment, the strength of the donor identity, and the experience at the deferral event.

First, the finding that not one woman (of five) who both worked and had dependent children returned within nine months of being eligible suggests that donors with the most responsibilities and demands on time are the most poorly placed to overcome the disruption to their donation pattern associated with deferral. This subset also tended to give blood on their own, not as part of an organised group. The following quotes are illustrative.

“Sometimes you could sit there for an hour before you were actually seen, and at that stage, you think you’re giving yourself enough time but the kids had to be picked up from school and something else, and so you’d sit there for an hour and then you couldn’t possibly wait any longer.” (Female, 47)

*“It’s just that busy stuff, like location, little kids, working, all those sorts of things, they were greater issues than* (needing to)… *rush out and give blood”(Female, 45)*

In contrast, the majority of older participants returned after deferral, even though they also tended to not give in an organised group. Fewer competing demands appeared to mean that return was less contingent on the activity remaining easy.

Second, younger donors without children seemed to have a greater chance of return if, prior to deferral, donation was facilitated by a supportive environment (e.g. giving blood in an organised group; a collection site convenient to work or home). Notions of convenience differed between younger donors and older donors, with one retired donor noting that blood donation was convenient even if he had to catch two buses to attend a collection site. In contrast, younger donors tended to report giving blood was easy if they lived or worked in the same suburb as a collection site or drove directly past one whilst commuting. In these ways, supportive structures appeared to facilitate return by reducing the effort needed to give blood.

The third attribute related to the strength of the donor identity. Returning donors tended to have strong self-perceptions of being a “blood donor”. This group saw donation working well in their lives: they found the activity personally rewarding, it was something that they could do with competence, and could be easily accommodated around their other commitments. Younger donors in particular saw blood donation in this way. When exploring the “blood donor” identity described by participants in this study, we found that role identity theory as traditionally applied to blood donor research such as [11-17] was a poor fit for our data. We considered an alternative conceptualisations of role identity that has not been previously applied to blood donors, that offered by McCall and Simmons (1978) [18]. This theory proposes that individuals have a role identity for each social position they occupy or wish to occupy. Successful role-performance, and the recognition of performance by others, is crucial in legitimising role-identities. Salience of a role identity (and therefore its likelihood of being enacted in a given situation) is influenced by four factors: its *prominence*; the need for external *support and recognition* of the identity; the need for the intrinsic and extrinsic *rewards* offered by enacting the identity; and the perceived opportunity for successful, *“profitable” enactment*[18].

Interestingly, many of those with a strong identity had previously encountered difficulties giving blood, such as unsatisfactory staff treatment, physical reactions, and deferrals, suggesting that repeated successful performance is only one contributing factor to the strength of the identity. For example, one young female participant had been deferred twice in her short donation career, and had in fact returned earlier than permitted after the first deferral, resulting in her being turned away. This donor appeared to derive numerous benefits from giving blood. She emphatically described her appreciation of the “rewards” of donation, such as the free health check, the social aspect of donation (as she attends with a large group of friends), the refreshments, the atmosphere at the donor centre, the way she was treated by the staff, and finally, positive self-perceptions as a result of knowing she was *“saving lives”*. Being a blood donor gave her a unique opportunity to demonstrate her compassion for others in a way that wasn’t otherwise possible within the time and economic constraints associated with her life stage. It is noteworthy that although this donor appeared to gain numerous benefits from being a blood donor, she was not alone in expressing a strong commitment to the activity despite experiencing problems at previous donation attempts.

The final attribute possibly related to non-return was unsatisfactory treatment at the deferral event. Of three donors who described aspects of unsatisfactory treatment, two did not return promptly, and the third was deceased at the time of follow up. One described a lack of compassion from the staff at the donor centre (“*I feel they don’t really care. I’m sure they don’t care”).* Another received a very brief explanation about her deferral, and felt that she was largely ignored by the nurses, which resulted in her feeling “*shafted*” and leaving the collection centre with many unanswered questions. The third also reported receiving *“snappy, abrupt”* treatment from the nursing staff. It is worth noting each member of this subset had other attributes found to be linked with a reduced likelihood of return.

Negative emotional responses have been proposed as a possible reason for the reduced likelihood of return after a temporary deferral [7,8]. However, the charting process identified few patterns between the ways participants reacted to their deferral and whether or not they returned. Patterns were also not found between donor return and length of donation history or other characteristics related to the donor (other than those described above).

### A conceptual model for explaining return from a temporary deferral

**Figure 3:**
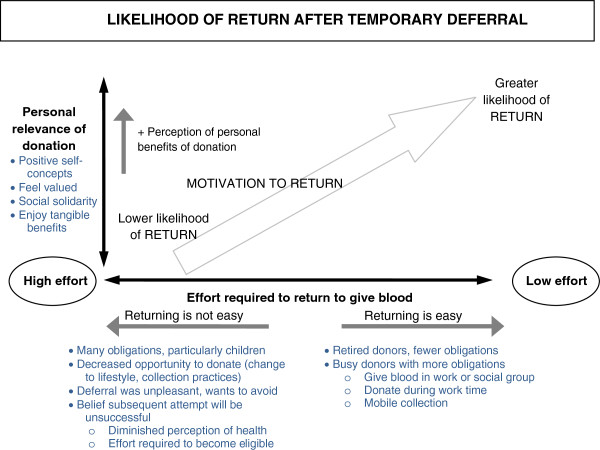
Conceptual model explaining likelihood of return after a temporary deferral for low haemoglobin.

The final stage of analysis, mapping and interpretation, resulted in the development of a conceptual model to explain why some donors returned promptly from deferral and others did not. The model is depicted in Figure 3.

Central to the model is the understanding that a deferral for a low haemoglobin level disrupts the habit of regular donation. This may be due, in part, to donors being unable to reinforce the strength of the association between context and donation behaviour during the six month deferral period [19]. The analysis also suggested that people are particularly vulnerable to interference from changes in their personal circumstances or collection practices when they are unable to give blood.

The horizontal axis in the figure represents the perceived convenience of donation. Perceived convenience is related to a number of factors: a donor’s lifestyle (such as work location); collection practices (such as collection site location or opening hours); obligations and demands in a donor’s life (such as family responsibilities); and whether attendance is facilitated by supportive environments. Assessments of convenience are also likely to be influenced by the practical and emotional “hassle” a deferral introduces to giving blood. Hassle incorporates the unpleasantness of the deferral event and the corresponding desire to avoid another occurrence, and reduced expectation of being accepted at a subsequent donation attempt.

The vertical axis in the figure represents the extent to which giving blood is personally relevant. As per McCall and Simmons’ conceptualisation of role identity [18], donors with strong role identities enjoyed the most benefits from donation. However, the salience of the blood donor role identity may diminish as a result of deferral. McCall and Simmons propose that salience of an identity is dependent, in part, on the opportunity for profitable enactment of the identity. Deferral may diminish donors’ expectation of successful subsequent donations, and furthermore, the mandatory six month deferral period means donors have no opportunity for profitable enactment for half a year, nor any contact from the blood service (beyond notification of test results in the week following deferral) that could serve to keep their identity in mind.

## Discussion

This study suggests that deferral reduces the likelihood of return through a number of processes. First, deferral disrupts the habit of regular donation, which also increases vulnerability to changes in personal circumstances and blood collection practices. Second, the deferral process is somewhat unpleasant and introduces a level of practical and emotional hassle to what was previously an undemanding activity. Third, deferral can diminish expectations that a future donation will be accepted, partly through reducing self-perceptions of good health and competence as a donor. In other words, deferral may “tip the scales” for a donor already juggling multiple demands, leading to the view that donation is too much of a hassle, particularly if the next attempt may be unsuccessful. Finally, the experience may reduce the strength of the blood donor identity if interpreted as unsuccessful role-performance, and limiting the opportunity for successful enactment. This analysis found relatively few differences between returning and non-returning donors in some respects: levels of altruism, length of donation history, knowledge of the need for blood, and experience of deferral (with the exception of those who felt particularly upset by their treatment). The findings of this study support the wider argument that opportunity for donation is the most important predictor of whether an individual gives blood, and that proximal factors, such as where and when to donate, should be the focus of recruitment efforts [20,21].

For re-engaging deferred donors, as well as recruitment of new donors, strategies to enhance convenience include maintaining a range of opening hours and locations (including mobile collections), and offering services such as transport and child-care arrangements. Another strategy endorsed by this research is supporting donation arrangements, such as through encouraging donation as part of a work or social group.

For deferred donors, aspects of the deferral event could be altered to reduce the practical and emotional hassle and enhance the perceived benefits. Better communication at the time of the donation attempt is clearly indicated and likely to be a key to overcoming several barriers to return. There may be an opportunity to offer a service during and following deferral that ensures donors feel that their health is important to the organisation, and that they are personally valued and appreciated. In addition to improved communication, strategies may include promoting the ferritin testing (that already occurs) as an additional benefit to the donor, and potentially, linking donors to other health services such as nutrition counselling and further investigations of the cause of low haemoglobin. Such a service might also maintain communication with deferred donors over the deferral period to increase the likelihood of return.

It has been recognised that in order to attract new donors, blood centre need to minimise perceived costs associated with donation [20]. Deferral introduces new costs for donors, such as unexpected news about their health, feelings of confusion, negative emotional responses, and perceptions of unsatisfactory staff treatment. Efforts need to be made to diminish these costs in order to allay fears about returning and a service such as that outlined would be one way to achieve this.

The literature on new forms of civil engagement proposes that volunteers are now less likely to form long-term commitments to a role or organisation, or engage in demanding commitments, compared to volunteers in the past [20,22,23]. The results of this study suggested that although donors are supportive of blood donation in principle, they are unable to commit much time or energy to the activity, and consequently, when giving blood becomes more difficult, or they believe attendance may not result in a successful contribution, the activity is less likely to be sustained. Those who find the activity personally relevant and rewarding appear to have the highest tolerance for disruption.

Paradoxically, blood services may be better placed to maintain community support than other voluntary organisations, as by its nature blood donation is sporadic, requires low levels of commitment, and is often performed alone. This study found deferred donors have high levels of good-will towards giving blood and the agencies responsible for its collection. The challenge for blood services is to recognise that current donors’ motivations and levels of commitment are different to those of previous generations [21], and to work to maintain the perception that blood donation is a good fit within individuals’ increasingly pressured lives.

In this regard, the work of Giddens is useful to appreciate that the biographical narratives informing self-identity are fragile [24]. If the continuity of a biography is vital for the integrity of self-perception, the movement from “I’m a capable blood donor” to “I have some problems giving blood” is likely to contribute to return being delayed after a deferral. Giddens also notes that particular behaviours reinforce other related role identities. This finding is supported in the current study, with several participants believing donation validates their good health, and that giving blood is a “natural” action for someone in their position. An inability to meet the minimum health standard required for blood donation could diminish the donors’ understandings of themselves as fit, healthy individuals, calling into question the assumption of a successful future attempt, and disrupting their perception of a natural relationship between good health and giving blood.

This study had some limitations. Due to the qualitative methodology and small, purposive sample size, results should be interpreted with caution, and regarded as provisional. Of note, only one individual deferred at first attempt to donate was interviewed, so findings largely relate to experienced donors. Also, only one participant was aged 25–39. All participants lived in the city so findings may not apply to rural donors (reached by a mobile service). It could be that individuals willing and able to be involved in this research had stronger “blood donor” identities and greater opportunities to accommodate donation into their lives than those who declined participation.

Participants were interviewed in the weeks immediately following deferral, in order to understand proximal responses to deferral and opportunities for improving the deferral process. Ideally, participants would have been interviewed again after return or non-return had been ascertained, but that was not feasible. It is also possible that interaction with the researcher may have changed the likelihood of return, as the interviews involved considerable reflection on the commitment to give blood and the deferral experience, and at least two additional contacts during the deferral period. It was not possible to identify all donors who were medically ineligible to return and some non-returning participants may have fallen into this category. Also, some participants may have returned more than nine months after being eligible to do so, therefore the research relates to relatively prompt return. The above limitations flow through to the conceptual model. Finally, the results presented in this article represent a degree of over-simplification of all possible patterns in the data. This was a consequence of tailoring analysis to answering a specific research question, which is a characteristic of Framework analysis.

## Conclusions

This study suggests deferral reduces the likelihood of return in a number of ways, relating the disruption of habit, the introduction of practical and emotion hassle to the donation process, and reduced strength of blood donor identity. The findings highlight the need to improve communication at the time of and following deferral, to enhance aspects of the deferral process to ensure individuals feel valued, and to maintain the convenience of giving blood to increase the likelihood of return.

## Competing interests

The authors declare that they have no conflicts of interest relevant to the manuscript submitted to BMC Public Health.

## Authors’ contributions

TH conceived of the study, developed the interview guide, carried out the interviews, performed the data analysis, and drafted the manuscript. KD contributed to analysis and helped draft the manuscript. VM participated in the design of the study, contributed to analysis and helped draft the manuscript. All authors read and approved the final manuscript.

## Pre-publication history

The pre-publication history for this paper can be accessed here:

http://www.biomedcentral.com/1471-2458/12/1063/prepub
